# Bacteria associated with *Amblyomma cajennense* tick eggs

**DOI:** 10.1590/S1415-475738420150040

**Published:** 2015

**Authors:** Erik Machado-Ferreira, Vinicius Figueiredo Vizzoni, Joseph Piesman, Gilberto Salles Gazeta, Carlos Augusto Gomes Soares

**Affiliations:** 1 Laboratório de Genética Molecular de Eucariontes e Simbiontes, Deptartamento de Genética, Instituto de Biologia, Universidade Federal do Rio de Janeiro, Rio de Janeiro, RJ, Brazil; 2 Laboratorio de Referência Nacional em Vetores das Riquetsioses, Instituto Oswaldo Cruz, Rio de Janeiro, RJ, Brazil; 3 Centers for Disease Control and Prevention, Division of Vector-Borne Diseases, Bacterial Diseases Branch, Fort Collins, CO, USA

**Keywords:** Amblyomma cajennense, tick eggs bacteria, Staphylococcus, Ixodidae

## Abstract

Ticks represent a large group of pathogen vectors that blood feed on a diversity of hosts. In the Americas, the Ixodidae ticks *Amblyomma cajennense* are responsible for severe impact on livestock and public health. In the present work, we present the isolation and molecular identification of a group of culturable bacteria associated with *A. cajennense* eggs from females sampled in distinct geographical sites in southeastern Brazil. Additional comparative analysis of the culturable bacteria from *Anocentor nitens, Rhipicephalus sanguineus* and *Ixodes scapularis* tick eggs were also performed. 16S rRNA gene sequence analyses identified 17 different bacterial types identified as *Serratia marcescens, Stenotrophomonas maltophilia, Pseudomonas fluorescens, Enterobacter* spp., *Micrococcus luteus, Ochrobactrum anthropi, Bacillus cereus* and *Staphylococcus* spp., distributed in 12 phylogroups. *Staphylococcus* spp., especially *S. sciuri,* was the most prevalent bacteria associated with *A. cajennense* eggs, occurring in 65% of the samples and also frequently observed infecting *A. nitens* eggs. *S. maltophilia, S. marcescens* and *B. cereus* occurred infecting eggs derived from specific sampling sites, but in all cases rising almost as pure cultures from infected *A. cajennense* eggs. The potential role of these bacterial associations is discussed and they possibly represent new targets for biological control strategies of ticks and tick borne diseases.

Ticks represent a large group of pathogen vectors that blood feed on a diversity of hosts, including amphibians, reptiles, birds and mammals. These Acari are divided into three families, Ixodidae (hard ticks), Argasidae (soft ticks), and the Nuttalliellidae (restricted to southern Africa) (Mihalca *et al.*, 2011). In the Americas, the Ixodidae ticks *Amblyomma cajennense* are responsible for the transmission and environmental maintenance of pathogens with public health relevance, including reports in southeastern Brazil (Lemos 2000; Walker *et al.*, 2000; Belongia, 2002; Krause, 2002; Galvão *et al.*, 2003; Guedes *et al.*, 2005; Guglielmone *et al.*, 2006). The Cayenne tick *A. cajennense* can transmit several pathogens to a variety of animals and is considered the main vector of *Rickettsia rickettsii*, the etiological agent of Rocky Mountain Spotted Fever (Guedes *et al.*, 2005).

Tick and tick borne disease control has been usually attempted or proposed by the use of a series of strategies aiming mostly on biological events to potentially impair tick feeding, pathogen transmission to mammal hosts (Allen and Humphreys, 1979; Kotsyfakis *et al.*, 2008; Piesman and Eisen, 2008; Rot *et al.*, 2013; Garcia *et al.*, 2014; Schwarz *et al.*, 2014), and in some cases, to restrict tick molting events and pathogen maintenance during tick development (Olds *et al.*, 2012; Calligaris *et al.*, 2013; Doan *et al.*, 2013; Moreno-Cid *et al.*, 2013; Rot *et al.*, 2013). Interestingly, studies on tick egg biology depicting possible strategies to improve egg health, integrity and development when exposed to natural environment, represent a poorly explored venue for the control of tick populations and associated pathogens. In fact, biological events, such as the possible role of the egg microbiome on tick population dynamics or tick development, to our knowledge has never been investigated and no bacteria were previously isolated specifically from tick eggs. However, many studies have described bacteria isolated from adult ticks (*Ixodes scapularis, Ixodes ricinus, Dermacentor reticulatus* and *Haemaphysalis concinna*) collected in the U.S., part of Europe and Australia (Martin and Schmidtmann, 1998; Murrell *et al.*, 2003; Stojek and Dutkiewicz, 2004; Rudolf *et al.*, 2009; Egyed and Makrai, 2014). In this scenario, we present the isolation and molecular identification of a group of culturable bacteria associated with *A. cajennense* eggs from females sampled in distinct geographical sites in southeastern Brazil. Comparative analyses with other Ixodidae ticks, such as *Anocentor nitens, Rhipicephalus sanguineus* and *I. scapularis* is presented. Natural colonization of tick eggs with specific bacterium physiotypes is discussed and it may bring new insights to the control of tick populations and tick-borne diseases.

In this work, actively feeding adult females of *A. cajennense* were retrieved from horses in southeastern Brazil sites, including municipalities with notified spotted fever cases (Brazil government data - *Ministério da Saúde*, SINAN, http://dtr2004.saude.gov.br/sinanweb/ from 2001 to 2015). Sampled ticks were maintained in laboratory until oviposition. Field collected samples of *A. nitens* and *R. sanguineus* actively feeding on horses and dogs were also obtained for comparative analyses. *I. scapularis* tick samples were also analyzed and obtained from U.S. colonies maintained at the Vector-Host Laboratory (Division of Vector-Borne Disease, Center for Disease Control and Prevention, CDC) (Table 1). Tick samples were prepared by initial washing steps, including four successive washes with ethanol 70% (v/v), followed by morphological and taxonomic confirmative analyses. Engorged females were placed in glass vials and maintained at ≈93% relative humidity, using a saturated solution of KNO_3_ in a growth chamber at 26 °C and under a photoperiod of 14 h: 10 h (light: dark) until oviposition. The obtained egg masses were washed in ethanol 70% (v/v), air-dried and homogenized in 500 μL of sterile PBS (phosphate buffered saline) with sterile mortar and pestle. Each egg mass, from a single female tick, was split in two to four sub-samples to generate independent egg/PBS homogenates which were individually plated for bacterial isolation.

**Table 1:** Bacterial isolates obtained from each tick egg mass sample analyzed.

Tick species	Sampling area/hosts	No. ticks laying eggs	Associated bacterial isolates		
			Identification^a^	Phylogroup (Clade)^b^	Prevalence^c^
*A. cajennense*	Três Rios, RJ, Brazil/horses	2	*Serratia* sp. Iso AC1 (EU693533)^d^ /100% - *Serratia marcescens* (KJ806487)	γ (B)	6/6
			*Stenotrophomonas* sp. Iso AC2 (EU693532)^e^ /100% - *Stenotrophomonas maltophilia* (KJ491015)	γ (D)	6/6
	Rio do Ouro, RJ, Brazil/horses	2	*Staphylococcus* sp. Iso AC3 (EU693530)^f^ /100% - *Staphylococcus sciuri* (KJ507203)	F (H)	3/4
			*Bacillus* sp. Iso AC4 (EU693531)^g^ /100% - *Bacillus cereus* (KJ534517)	F (G)	4/4
	Pouso Alto, MG, Brazil/horses	2	*Staphylococcus* sp. Iso AC5 (KP306739) /100% - *Staphylococcus sciuri* (KJ507203)	F (H)	4/4
	Seropédica, RJ, Brazil/horses	2	*Staphylococcus* sp. Iso AC6 (KP306740) /100% - *Staphylococcus kloosii* (JX102547)	F (I)	3/6
			*Staphylococcus* sp. Iso AC7 (KP306741) /100% - *Staphylococcus agnetis* (HM484986)	F (K)	6/6
			*Staphylococcus* sp. Iso AC8 (KP306742) /99% - *Staphylococcus aureus* (HM559234)	F (L)	6/6
*A. nitens*	Seropédica, RJ, Brazil / horses	3	*Staphylococcus* sp. Iso AN1 (KP306743) /99% - *Staphylococcus sciuri* (KJ507203)	F (H)	8/8
			*Staphylococcus* sp. Iso AN2 (KP306745) /99% - *Staphylococcus saprophyticus* (KF254616)	F (J)	8/8
			*Enterobacter* sp. Iso AN3 (KP306734) /99% - *Enterobacter aerogenes* (KJ631293)	γ (A)	5/8
*R. sanguineus*	Boa Esperança, MG, Brazil/dogs	3	*Pseudomonas* sp. Iso RS1 (KP306738) /100% - *Pseudomonas fluorescens* (AB680296)	γ (C)	2/4
			*Enterobacter* sp. Iso RS2 (KP306735) /99% - *Enterobacter hormaechei* (KF054945)	γ (A)	4/4
*I. scapularis*	Laboratory-reared, USA	4	*Stenotrophomonas* sp. Iso IS1 (KP306746) /99% - *Stenotrophomonas maltophilia* (JF681290)	γ (D)	14/14
			*Stenotrophomonas* sp. Iso IS2 (KP306744) /100% - *Stenotrophomonas maltophilia* (JF681290)	γ (D)	12/14
			*Micrococcus* sp. Iso IS3 (KP306736) /100% - *Micrococcus luteus* (JX262404)	A (F)	8/14
			*Ochrobactrum* sp. Iso IS4 (KP306737) /100% - *Ochrobactrum anthropi* (KF956631)	α (E)	4/14
Total		18			46

a Identification by 16S rRNA gene sequence analyses. The GenBank accession number is given in parenthesis. The identity percentage with the closest species is followed by slash; The most identical species name and GenBank accession number is also presented.

b Phylogroup as follows: A= Actinobacteria; F = Firmicutes; α = Alpha-Proteobacteria; γ = Gamma-Proteobacteria. Clade code “A” to “N” refers to the clusters in the 16S rRNA gene based phylogenetic reconstruction presented in the Figure 1.

c Prevalence= No. positive egg-PBS suspension samples for a specific bacterial isolate/ Total No. of egg_PBS samples analyzed. Each egg mass from a single female tick was splitted in 2 to 4 samples to generate independent egg_PBS homogenates. See text for details.

d Previously annotated as *Serratia marcescens* strain CS265 in the Genbank.

e Previously annotated as *Stenotrophomonas maltophilia* strain CS266 in the Genbank.

f Previously annotated as *Staphylococcus sciuri* strain CS264 in the Genbank.

g Previously annotated as *Bacillus cereus* strain CS262 in the Genbank.

Bacteria were isolated by direct streaking of 50 μL of the egg/PBS suspensions on LB (Luria-Bertani medium) agar plates, without any enrichment step to prevent competitive selection to occur. Replicas were incubated for 24 h at 30 °C and 37 °C. Bacterial colonies with the same morphology were purified in triplicates and directly inoculated into liquid LB medium for subsequent DNA extraction and storage at −80 °C. Bacterial genomic DNA extraction was routinely performed using the DNeasy Blood and Tissue Kit (Qiagen, Germantown, MD) procedure and eluted in 100 μL of TE buffer. DNA integrity was analyzed by agarose gel electrophoresis (data not shown).

The eubacterial 16S rRNA universal primers 27f, 338f, 907r and 1492r (Lane, 1991) were applied in PCR reactions using the purified bacterial genomic DNAs. PCR conditions included 35 cycles of 94 °C for 1 min, 55 °C for 35 s and 72 °C for 1 min. PCR products from four independent reactions were directly purified with the GFX PCR DNA and Gel band purification kit (GE Healthcare, Buckinghamshire, UK), according to manufacturer's instructions. Purified PCR products were sequenced using the BigDye Terminator DNA sequencing kit (Applied Biosystems, Foster City, Calif., U.S.A.) and analyzed in a Megabace 1000 DNA sequencer (Amersham Biosciences). Sequences were edited using SeqMan program (DNASTARinc package for Windows platform, 1989-1999), and the identities were obtained by BLAST analyses. Neighbor-joining phylogenetic reconstruction with the Kimura two-parameter correction model was used to obtain a better taxonomic resolution (Kimura, 1980).

A total of 72 morphologically different colonies were visually grouped and isolated from a total of 46 egg mass samples, including 20 samples analyzed for *A. cajennense* from 4 sampling sites located in the states of Rio de Janeiro and Minas Gerais, Brazil, and 26 comparative samples of egg masses from *A. nitens, R. sanguineus* and *I. scapularis* species. DNA sequence BLAST analyses grouped all isolates into a total of 17 bacterial types (named as “IsoAC1” to “IsoAC8”, “IsoAN1” to “IsoAN3”, “IsoRS1” and “IsoRS2”, “IsoIS1” to “IsoIS4”) belonging to eight bacterial genera (Table 1), distributed in 12 phylogroups of *Firmicutes* (Bacilli) (70% of *A. cajenennse* samples; ~47% of all samples), *Gamma-proteobacteria* (30% of *A. cajennense* samples; ~42% of all samples), *Alpha-proteobacteria* (not in *A. cajennense* but in ~5,5% of all samples) and *Actinobacteria* (not in *A. cajennense* but in ~5,5% of all samples) after 16S rRNA gene sequence analyses (Figure 1). Curiously, most tick egg samples present a restricted culturable bacterial richness, especially *A. cajennense* samples which in some cases yielded isolation of a pure culture of the associated *Staphylococcus sciuri* by direct plating of its egg/PBS homogenates. *Staphylococcus* was the most frequent genus of bacteria associated with all tick species tested, occurring in 65% of the *A. cajenennse* samples and 45% of all tested egg/PBS homogenates, especially the *S. sciuri* (Clade H), which occurred in tick eggs sampled in different states of Brazil, both from Cayenne ticks sampled in the cities of Rio do Ouro (Rio de Janeiro state) and Pouso Alto (Minas Gerais state), and from *A. nitens* obtained in Seropedica (Rio de Janeiro state). *Staphylococcus* spp. were also abundantly detected in a recent metagenomic assessment of bacteria in *R. microplus*, but *S. sciuri* was only found associated to adult ticks of this species (Andreotti *et al.*, 2011). Similarly to the isolates cultured from *A. cajennense* eggs, these authors also detected *S. aureus*, other *Staphylococcus* spp., *Serratia marcescens, Stenotrophomonas* sp. and *Pseudomonas* sp. in *R. microplus* egg samples.

**Figure 1:**
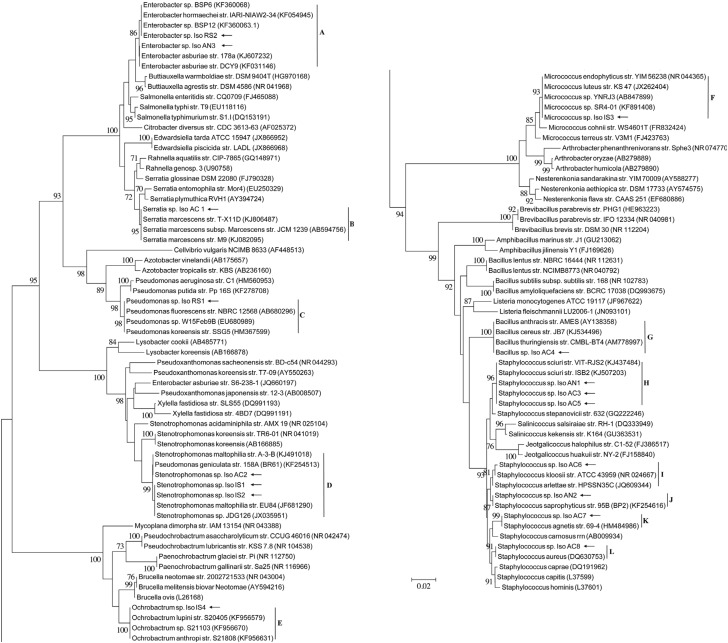
Phylogenetic inference of the tick egg associated bacteria using 16S rRNA gene sequences. Neighbor-Joining analysis with Kimura 2-parameter based on the nucleotide sequences was performed. *Arrows* indicate sequences obtained in the present work, and the GenBank accession codes for other sequences are presented in parenthesis. Sequences were aligned using the ClustalW program (Promega, Madison, WI), and phylogenetic inferences obtained using the MEGA 5.2.2 software. Internal node supports were calculated using bootstrap analyses with 1,000 replicas. Bootstrap values below 70% are not present.

*Serratia marcescens* (Clade B) and *Stenotrophomonas maltophilia* (Clade D) were the only *Gamma-Proteobacteria* isolated from *A. cajennense* egg samples and observed coinfecting the eggs from females collected in the Três Rios site. In addition, members of *Stenotrophomonas* sp. (Clade D) and phylogenetically close to the Cayenne tick isolates, were also observed in association with *I. scapularis* in the U.S.

Ticks can uptake and carry bacteria from their environment or from the skin surface of hosts and some of these bacteria are able to survive and replicate in ticks (Egyed and Makrai, 2014). *S. sciuri* is in fact considered common colonizers of dogs, cats, found in the skin of cattle and other animals (Devriese *et al.*, 1984; Cox *et al.*, 1985; Lilenbaum *et al.*, 1999; Bagcigil *et al.*, 2007; Andreotti *et al.*, 2011; Couto *et al.*, 2011; Garbacz *et al.*, 2013). In this work, several identified bacteria are not typically described as associated with arthropods. The successive washes of the female ticks before oviposition, as previously performed in other tick associated bacteria study (Jutras *et al.*, 2010), and taking the fact that eggs were laid in the laboratory vials and subjected to an additional direct washing step, suggests that these bacteria are not casual soil or other environmental population accidently colonizing the tested egg samples, and could indeed represent active partners participating in specific aspects of tick physiology. It is interesting to mention that all bacterial isolates are able to secrete protease, as determined by clearing zone formation by growths on LB agar plates containing 0.5% casein (Supplementary Material, Figure S1). This suggests that all isolated bacteria could contribute to ticks egg hatching processes, which should be assessed in further work. Also, a growing literature indicates that arthropods containing associated bacteria increase the arthropod-resistance against parasites and/or pathogens (Gravot *et al.*, 2000; Oliver *et al.*, 2003; Hedges and Johnson, 2008; Teixeira *et al.*, 2008; Brownlie and Johnson, 2009; Koehler and Kaltenpoth, 2013; Koehler *et al.*, 2013). The exposed tick eggs are vulnerable to environmental conditions and infection, mostly by soil microorganisms, is expected. Although not elucidated in the present work, we can speculate that the reduced amount of bacterial types isolated from the eggs may indicate that those bacteria have outcompeted other bacterial types in these samples. If somehow ticks manage to select specific egg colonizing bacteria and mostly maintain bioactive metabolite producing bacteria, it would in fact represent a strategy for chemical defense to improve success of egg development, hatching and tick population fitness. Interestingly, most isolated bacterial species are members of phyloclades that include known antifungal, bacteriocins and bioactive metabolites-producing bacteria, such as *S. sciuri* (Clade H), *S. maltophilia* (Clade D), *S. marcescens* (Clade B), *P. fluorescens* (Clade C), *M. luteus* (Clade F), *S. aureus* (Clade L), *B. cereus/B. thuringiensis* (Clade G) (Gardner, 1949; Tagg *et al.*, 1976; Jakobi *et al.*, 1996; Harris *et al.*, 2004; Furushita *et al.*, 2005; Pankewitz and Hilker, 2006; Banerjee *et al.*, 2011; Gutiérrez-Román *et al.*, 2012; Liu *et al.*, 2013), indicating their possible role on chemical protection of the exposed egg masses. *S. marcensces* and *B. cereus* are also known to combat insects and nematodes, organisms that could represent predators of tick eggs (Sikorowski and Lawrence, 1998; Yoshida *et al.*, 2001; Dillon and Charnley, 2002; Nishiwaki *et al.*, 2004). Additionally, the most frequent *S. sciuri* and *S. maltophilia* are also known as multidrug resistant bacteria (Stepanovic *et al.*, 2006; Hauschild *et al.*, 2007; Haenni *et al.*, 2011; Bhargava and Zhang, 2012; Lozano *et al.*, 2012; Huang *et al.*, 2013; Davis *et al.*, 2014; García-León *et al.*, 2014; Harrison *et al.*, 2014) and that would possibly support their high prevalence on *A. cajennense* egg samples, as a consequence of their potential resistance to competing microorganisms or to other antimicrobial components on eggs surface or in the surrounding environment (Arrieta *et al.*, 2006).

Some bacterial species isolated are also known to be pathogenic to arthropod hosts, as the Clade G *B. cereus/B. thuringiensis*. The Clade B *S. marcensces* also seems to be harmful to ticks, since it was already described that the wax from the eggs of *Amblyomma hebraeum* has antibiotic activity for their protection specifically against this bacterium (Arrieta *et al.*, 2006). Nymphs, larvae and adult ticks of *Amblyomma variegatum* also present specific substances against *S. marcensces* (Pavis *et al.*, 1994). This bacterium species is a known opportunistic pathogen of insects (Sikorowski and Lawrence, 1998) and able to decrease egg hatching time of flies (Romero *et al.*, 2006). Some other bacterial isolates are also representatives of clades that include potential pathogens to ticks, and it raises the possibility that tick egg associated bacteria could also act on trans-generational immune priming, a process of maternal transfer of bacteria to increase the expression of immunity-related genes encoding antibacterial proteins in the emerging larvae, as described for some insects (Freitak *et al.*, 2014). These possibilities should be tested in the future.

It is important to mention that the bacteria presented here were cultured and isolated, not only described at the DNA sequence level. These bacteria, specially *S. sciuri*, are potential candidates for future paratransgenesis strategies, once they were easily isolated from tick eggs and reported infecting both nymph and adult ixodidae ticks of different species, including *R. microplus, Ixodes holocyclus, I. ricinus, Dermacentor reticulatus, Haemaphysalis concina* and *Amblyomma fimbriatum* (Martin and Schmidtmann, 1998; Murrell *et al.*, 2003; Stojek and Dutkiewicz 2004; Rudolf *et al.*, 2009; Andreotti *et al.*, 2011). In paratransgenesis, arthropod associated bacteria are used as vehicles for expressing foreign genes to kill or reduce pathogen fitness in their arthropod vectors, representing an alternative strategy to reduce pathogen transmission (Beard *et al.*, 1998). Taken together, the present description and isolation of tick eggs associated bacteria offer new targets and tools for biological control strategies of ticks and tick borne diseases.

## Supplementary Material

The following online material is available for this article:

Figure S1 - Detection of protease production by the tick eggs bacterial isolates

This material is available as part of the online article from http://www.scielo.br/gmb.

## References

[B1] Allen JR, Humphreys SJ (1979). Immunisation of guinea pigs and cattle against ticks. Nature.

[B2] Andreotti R, Leon AAP de, Dowd SE, Guerrero FD, Bendele KG, Scoles GA (2011). Assessment of bacterial diversity in the cattle tick Rhipicephalus (Boophilus) microplus through tag-encoded pyrosequencing. BMC Microbiol.

[B3] Arrieta MC, Leskiw BK, Kaufman WR (2006). Antimicrobial activity in the egg wax of the African cattle tick Amblyomma hebraeum (Acari, Ixodidae). Exp Appl Acarol.

[B4] Bagcigil FA, Moodley A, Baptiste KE, Jensen VF, Guardabassi L (2007). Occurrence, species distribution, antimicrobial resistance and clonality of methicillin- and erythromycin-resistant staphylococci in the nasal cavity of domestic animals. Vet Microbiol.

[B5] Banerjee D, Chatterjee S, Banerjee UC, Guha AK, Ray L (2011). Green pigment from Bacillus cereus M(1)(16) (MTCC 5521): Production parameters and antibacterial activity. Appl Biochem Biotechnol.

[B6] Beard CB, Durvasula RV, Richards FF (1998). Bacterial symbiosis in arthropods and the control of disease transmission. Emerg Infect Dis.

[B7] Belongia EA (2002). Epidemiology and impact of coinfections acquired from Ixodes ticks. Vector Borne Zoonotic Dis.

[B8] Bhargava KL, Zhang Y (2012). Multidrug-resistant coagulase-negative Staphylococci in food animals. J Appl Microbiol.

[B9] Brownlie JC, Johnson KN (2009). Symbiont-mediated protection in insect hosts. Trends Microbiol.

[B10] Calligaris IB, De Oliveira PR, Roma GC, Bechara GH, Camargo-Mathias MI (2013). Action of the insect growth regulator fluazuron, the active ingredient of the acaricide Acatak, in Rhipicephalus sanguineus nymphs (Latreille, 1806) (Acari, Ixodidae). Microsc Res Tech.

[B11] Couto N, Pomba C, Moodley A, Guardabassi L (2011). Prevalence of meticillin-resistant Staphylococci among dogs and cats at a veterinary teaching hospital in Portugal. Vet Rec.

[B12] Cox HU, Hoskins JD, Newman SS, Turnwald GH, Foil CS, Roy AF, Kearney MT (1985). Distribution of staphylococcal species on clinically healthy cats. Am J Vet Res.

[B13] Davis JA, Jackson CR, Ferdorka-Cray PJ, Barrett JB, Brousse JH, Gustafson J, Kucher M (2014). Carriage of methicillin-resistant staphylococci by healthy companion animals in the US. Lett Appl Microbiol.

[B14] Devriese LA, Nzuambe D, Godard C (1984). Identification and characterization of staphylococci isolated from cats. Vet Microbiol.

[B15] Dillon R, Charnley K (2002). Mutualism between the desert locust Schistocerca gregaria and its gut microbiota. Res Microbiol.

[B16] Doan HT, Noh JH, Kim YH, Yoo MS, Reddy KE, Jung SC, Kang SW (2013). The efficacy of avermectins (ivermectin, doramectin and abamectin) as treatments for infestation with the tick Haemaphysalis longicornis on rabbits in Korea. Vet Parasitol.

[B17] Egyed L, Makrai L (2014). Cultivable internal bacterial flora of ticks isolated in Hungary. Exp Appl Acarol.

[B18] Freitak D, Schmidtberg H, Dickel F, Lochit G, Vogel H, Vilcinskas A (2014). The maternal transfer of bacteria can mediate trans-generational immune priming in insects. Virulence.

[B19] Furushita M, Okamoto A, Maeda T, Ohta M, Shiba T (2005). Isolation of multidrug-resistant Stenotrophomonas maltophilia from cultured yellowtail (Seriola quinqueradiata) from a marine fish farm. Appl Environ Microbiol.

[B20] Galvão MA, Mafra CL, Moron C, Anaya E, Walker DH (2003). Rickettsiosis of the genus Rickettsia in South America. Ann NY Acad Sci.

[B21] Garbacz K, Zarnowska S, Piechowicz L, Haras K (2013). Staphylococci isolated from carriage sites and infected sites of dogs as a reservoir of multidrug resistance and methicillin resistance. Curr Microbiol.

[B22] Garcia GR, Gardinassi LG, Ribeiro JM, Anatriello E, Ferreira BR, Moreira HN, Mafra C, Martins MM, Szabó MP, de Miranda-Santos IK (2014). The sialotranscriptome of Amblyomma triste, Amblyomma parvum and Amblyomma cajennense ticks, uncovered by 454-based RNA-seq. Parasit Vectors.

[B23] Garcia-Leon G, Salgado F, Oliveros JC, Sanchez MB, Martinez JL (2014). Interplay between intrinsic and acquired resistance to quinolones in Stenotrophomonas maltophilia. Environ Microbiol.

[B24] Gardner JF (1949). An antibiotic produced by Staphylococcus aureus. Br J Exp Pathol.

[B25] Gravot E, Thomas-Orillard M, Jeune B (2000). Virulence variability of the Drosophila C virus and effects of the microparasite on demographic parameters of the host (Drosophila melanogaster). J Invertebr Pathol.

[B26] Guedes E, Leite RC, Prata MC, Pacheco RC, Walker DH, Labruna MB (2005). Detection of Rickettsia rickettsii in the tick Amblyomma cajennense in a new Brazilian spotted fever-endemic area in the state of Minas Gerais. Mem Inst Oswaldo Cruz.

[B27] Guglielmone AA, Beati L, Barros-Battesti DM, Labruna MB, Nava S, Venzal JM, Mangold AJ, Szabo MP, Martins JR, Gonzalez-Acuna D (2006). Ticks (Ixodidae) on humans in South America. Exp Appl Acarol.

[B28] Gutiérrez-Román MI, Holguín-Meléndez F, Bello-Mendoza R, Guillén-Navarro K, Dunn MF, Huerta-Palacios G (2012). Production of prodigiosin and chitinases by tropical Serratia marcescens strains with potential to control plant pathogens. World J Microbiol Biotechnol.

[B29] Haenni M, Châtre P, Boisset S, Carricajo A, Bes M, Laurent F, Madec JY (2011). Staphylococcal nasal carriage in calves: multiresistant Staphylococcus sciuri and immune evasion cluster (IEC) genes in methicillin-resistant Staphylococcus aureus ST398. J Antimicrob Chemother.

[B30] Harris AK, Williamson NR, Slater H, Cox A, Abbasi S, Foulds I, Simonsen HT, Leeper FJ, Salmond GPC (2004). The Serratia gene cluster encoding biosyntesis of the red antibiotic, prodigiosin, shows species-and strain-dependent genome context variation. Microbiology.

[B31] Harrison EM, Paterson GK, Holden MT, Ba X, Rolo J, Morgan FJ, Pichon B, Kearns A, Zadoks RN, Peacock SJ (2014). A novel hybrid SCCmec-mecC region in Staphylococcus sciuri. J Antimicrob Chemother.

[B32] Hauschild T, Vukovic D, Dakic I, Jezek P, Djukic S, Dimitrijevic V, Stepanovic S, Schwarz S (2007). Aminoglycoside resistance in members of Staphylococcus sciuri group. Microb Drug Resist.

[B33] Hedges LM, Johnson KN (2008). The induction of host defence responses by Drosophila C virus. J Gen Virol.

[B34] Huang YW, Hu RM, Yang TC (2013). Role of the pcm-tolCsm operon in the multidrug resistance of Stenotrophomonas maltophilia. J Antimicrob Chemother.

[B35] Jakobi M, Winkelmann G, Kaiser D, Kempler C, Jung G, Berg G, Bahl H (1996). Maltophilin: a new antifungal compound produced by Stenotrophomonas maltophilia R3089. J Antibiot.

[B36] Jutras BL, Liu Z, Brissette CA (2010). Simultaneous isolation of Ixodidae and bacterial (Borrelia spp.) genomic DNA. Curr Protoc Microbiol.

[B37] Kimura M (1980). A simple method for estimating evolutionary rates of base substitutions through comparative studies of nucleotide sequences. J Mol Evol.

[B38] Koehler S, Kaltenpoth M (2013). Maternal and environmental effects on symbiont-mediated antimicrobial defence. J Chem Ecol.

[B39] Koehler S, Doubsky J, Kaltenpoth M (2013). Dynamics of symbiont-mediated antibiotic production reveal efficient long-term protection for beewolf offspring. Front Zool.

[B40] Kotsyfakis M, Anderson JM, Andersen JF, Calvo E, Francischetti IM, Mather TN, Valenzuela JG, Ribeiro JM (2008). Cutting edge: Immunity against a “silent” salivary antigen of the Lyme vector Ixodes scapularis impairs its ability to feed. J Immunol.

[B41] Krause PJ (2002). Babesiosis. Med Clin North Am.

[B42] Lane DJ, Stackebrandt E, Goodfellow M (1991). 16S/23S rRNA sequencing. Nucleic Acid Techniques in Bacterial Systematics.

[B43] Lemos ERS (2000). Rickettsial diseases in Brazil. Virus Rev Res.

[B44] Lilenbaum W, Esteves AL, Souza GN (1999). Prevalence and antimicrobial susceptibility of staphylococci isolated from saliva of clinically normal cats. Lett Appl Microbiol.

[B45] Liu J, Chen P, Zheng C, Huang YP (2013). Characterization of maltocin P28, a novel phage tail-like bacteriocin from Stenotrophomonas maltophilia. Appl Environ Microbiol.

[B46] Lozano CL, Aspiroz C, Sáenz Y, Ruiz-García M, Royo-García G, Gómez-Sanz E, Ruiz-Larrea F, Zarazaga M, Torres C (2012). Genetic environment and location of the lnu(A) and lnu(B) genes in methicillin-resistant Staphylococcus aureus and other staphylococci of animal and human origin. J Antimicrob Chemother.

[B47] Martin PA, Schmidtmann ET (1998). Isolation of aerobic microbes from Ixodes scapularis (Acari, Ixodidae), the vector of Lyme disease in the eastern United States. J Econ Entomol.

[B48] Mihalca AD, Gherman CM, Cozma V (2011). Coendangered hard-ticks: Threatened or threatening?. Parasit Vectors.

[B49] Moreno-Cid JA, Pérez de la Lastra JM, Villar M, Jiménez M, Pinal R, Estrada-Peña A, Molina R, Lucientes J, Gortázar C, Fuente J de la, SUB/AKR Vaccine Study Group (2013). Control of multiple arthropod vector infestations with subolesin/akirin vaccines. Vaccine.

[B50] Murrell A, Dobson SJ, Yang X, Lacey E, Barker SC (2003). A survey of bacterial diversity in ticks, lice and fleas from Australia. Parasitol Res.

[B51] Nishiwaki H, Ito K, Otsuki K, Yamamoto H, Komai K, Matsuda K (2004). Purification and functional characterization of insecticidal sphygomyelinase C produced by Bacillus cereus. Eur J Biochem.

[B52] Olds C, Mwaura S, Crowder D, Odongo D, Oers M van, Owen J, Bishop R, Daubenberger C (2012). Immunization of cattle with Ra86 impedes Rhipicephalus appendiculatus nymphal-to-adult molting. Ticks Tick Borne Dis.

[B53] Oliver KM, Russell JA, Moran NA, Hunter MS (2003). Facultative bacterial symbionts in aphids confers resistance to parasitic wasps. Proc Natl Acad Sci USA.

[B54] Pankewitz F, Hilker M (2006). Defensive components in insect eggs: are anthraquinones produced during egg development?. J Chem Ecol.

[B55] Pavis C, Mauleon H, Barre N, Maibeche M (1994). Dermal gland secretions of tropical bont tick, Amblyomma variegatum (Acarina, Ixodidae) Biological activity on predators and pathogens. J Chem Ecol.

[B56] Piesman J, Eisen L (2008). Prevention of tick-borne diseases. Annu Rev Entomol.

[B57] Romero A, Broce A, Zurek L (2006). Role of bacteria in the ovoposition behaviour and larval development of stable flies. Med Vet Entomol.

[B58] Rot A, Gindin G, Ment D, Mishoutchenko A, Glazer I, Samish M (2013). On-host control of the brown dog tick Rhipicephalus sanguineus Latreille (Acari, Ixodidae) by Metarhizium brunneum (Hypocreales, Clavicipitaceae). Vet Parasitol.

[B59] Rudolf I, Mendel J, Sikutova S, Svec P, Masaryková J, Novaková D, Bunkova L, Sedlacek I, Hubalek Z (2009). 16S rRNA gene-based identification of cultured bacterial flora from host-seeking Ixodes ricinus, Dermacentor reticulatus and Haemaphysalis concinna ticks, vectors of vertebrate pathogens. Folia Microbiol.

[B60] Schwarz A, Tenzer S, Hackenberg M, Erhart J, Gerhold-Ay A, Mazur J, Kuharev J, Ribeiro JM, Kotsyfakis M (2014). A systems level analysis reveals transcriptomic and proteomic complexity in Ixodes ricinus midgut and salivary glands during early attachment and feeding. Mol Cell Proteomics.

[B61] Sikorowski PP, Lawrence AM (1998). Transmission of Serratia marcescens (Enterorobacteriaceae) in adult Heliothis virescens (Lepidoptera, Noctuidae) laboratory colonies. Biol Control.

[B62] Stepanovic S, Martel A, Dakic I, Decostere A, Vukovic D, Ranin L, Devriese LA, Haesebrouck F (2006). Resistance to macrolides, lincosamides, streptogramins, and linezolid among members of Staphylococcus sciuri group. Microb Drug Resist.

[B63] Stojek NM, Dutkiewicz J (2004). Studies on the occurrence of Gram-negative bacteria in ticks: Ixodes ricinus as a potential vector of Pasteurella. Ann Agric Environ Med.

[B64] Tagg JR, Dajani AS, Wannamaker LW (1976). Bacteriocins of gram-positive bacteria. Bacteriol Rev.

[B65] Teixeira L, Ferreira A, Ashburner M (2008). The bacterial symbiont Wolbachia induces resistance to RNA viral infections in Drosophila melanogaster. PLoS Biol.

[B66] Walker JB, Keirans JE, Horak IG (2000). The Genus Rhipicephalus (Acari, Ixodidae): A Guide to the Brown Ticks of the World.

[B67] Yoshida N, Oeda K, Watanabe E, Mikame T, Fukita Y, Nishimura K, Komai K, Matsuda K (2001). Chaperonin turned insect toxin. Nature.

