# Supplementary material for: Bacteria associated with Amblyomma cajennense tick eggs
Source: Genet Mol Biol. 2015 Oct-Dec;38(4):477–83. doi: 10.1590/S1415-475738420150040 (PMC4763323; doi:10.1590/S1415-475738420150040)
Supplement: Figure S1 - [file 1415-4757-gmb-S1415-475738420150040-s001.pdf]

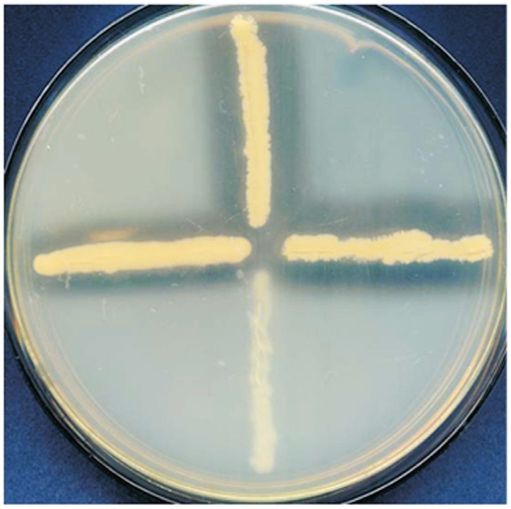

Figure S1 - Detection of protease production by the tick eggs bacterial isolates *Stenotrophomonas* sp. Iso AC2, *Staphylococcus* sp. Iso AC3 and *Bacillus* sp. Iso AC4. This is a representative figure for all tick eggs isolates. An *E. coli* negative control was included. Bacteria were grown on LB agar covered with LB + 0.5% casein top agar. Plates were incubated at 30 °C/24 h, followed by a 0.1N HCl washing step for a better visualization of the degraded casein halo formation. See article *Table 1* for details on the strains isolation.
